# Canine Food Preference Assessment of Animal and Vegetable Ingredient-Based Diets Using Single-Pan Tests and Behavioral Observation

**DOI:** 10.3389/fvets.2017.00154

**Published:** 2017-10-03

**Authors:** Meghan C. Callon, Cara Cargo-Froom, Trevor J. DeVries, Anna K. Shoveller

**Affiliations:** ^1^Department of Animal Biosciences, University of Guelph, Guelph, ON, Canada

**Keywords:** food preference, canine, animal-based protein, vegetable-based protein, single-pan, neophobia, pet food

## Abstract

Knowledge of canine food selection is critical for both the pet food industry and dog owners, since owners want quality foods that are palatable, while fulfilling their pet’s nutritional requirements. There are two common methods for assessing canine food preference: the two-pan test and the one-pan test. Neither test fully accounts for the complexity of the canine feeding experience nor do they provide applicable representations of canine feeding behavior in the home. The objectives of this study were to (1) determine whether dogs display a preference for animal ingredient-based diets when compared with vegetable ingredient-based diets and (2) examine whether dogs experience neophobia when presented with a novel diet. Eight adult Beagles (average age = 24 months, weighing 8–12 kg) were individually fed each of four novel diets in a 4 × 4 replicated Latin square design, with 10-d treatment periods and four dietary treatments. Data were analyzed using a mixed model with repeated measures and significance was declared when *p* < 0.05. The diets were: animal and vegetable ingredient-based diets, and animal- and vegetable-based ingredients diluted with anhydrous α-d-glucose. The diluted diets were used for a larger study to determine true mineral digestibility. Dogs were fed twice per day (0800 and 1300 h). Behavioral observations were made by video on the first, and last 2 days of each 10-day treatment period of both a.m. and p.m. feedings. Time to consume feed, distraction, hesitation, level of anticipation pre-consumption, and interest post-consumption were recorded. Dogs experienced initial disruptive (neophobic) effects of a novel diet. Neophobia was demonstrated by a decreased (slower) rate of consumption, increased distraction during consumption of the diet, and increased hesitation on the first day of each new diet (*p* < 0.05). The level of interest post-consumption was highest when dogs consumed the animal-based ingredients diet (*p* < 0.05). This study presents insights into canine food preference assessment methods that may more accurately represent the dog owner’s experience. Further research is required to determine the minimum length of time necessary to eliminate neophobia to food. In addition, future research should also aim to establish whether interest post-consumption is due primarily to food preference or acute satiety.

## Introduction

The study of canine food selection is crucial for both the pet food industry and dog owners. Pet owners want quality dog foods that fulfill their pet’s nutritional requirements, as well as being palatable and multiple approaches to assessing this have been reviewed (1). It is generally accepted by the industry that the top reasons for dog owners to switch food is: their dog disliked the previous food, price, and addressing a certain health outcome (i.e., skin and coat). The likelihood of consumption of a food source comes down to palatability, or the subjective preference of a food based on odor, texture, appearance, and taste (2, 3). In dogs, food preference is influenced both by early-life experiences and genetics (4). Health status, age, and environmental conditions may all influence an animal’s perception of a food source (5). Together, these factors influence the perceived palatability of a food source and subsequent feeding behavior.

Since animals cannot directly communicate to indicate their food preferences, measurements of food preference are assessed by comparing relative acceptance of different diets. The two most common methods to assess food preference in dogs are the two-pan test and one-pan test (6). The two-pan, or split plate, test consists of presenting two different food sources to the animal and recording the amount consumed of each (6). The one-pan test often involves free-feeding one food source at a time, recording the amount of food consumed over a specified period, and then comparing that to one or more other feed types.

A one-pan test provides a more controlled method of assessing a dog’s initial reaction to a novel food source, as well as measuring any effects which occur due to a dietary change. It also eliminates any food interactions, where one may alter the palatability of the other, which may occur during a two-pan test (6). However, neither of the traditional one- or two-pan tests control for the effects of satiety. It is proposed that a one-pan test with controlled amounts of food may not only provide an accurate view of an animal’s food preference, but also be a more applicable representation of canine feeding behavior in the home. Using a one-pan test, with limited food availability, provides an opportunity to closely examine select behaviors that accompany the canine feeding experience. This is the first study to take this approach to examine dog feeding behavior; however, similar approaches have been utilized in other animals [cats (7); rats (8); human and non-human primates (9)].

Another key concept that influences canine feeding behavior is that of neophobia. Stöwe et al. (10) describe neophobia as “the avoidance of an object or other aspect of the environment solely because it has never been experienced and is dissimilar from what has been experienced in the individual’s past.” Although dogs are considered to be naturally neophilic, which is a preference for novelty, neophobia has been frequently encountered with respect to novel food sources (11). Neophobic animals are often slow to investigate a novel object or food source and demonstrate reduced attentiveness to the task (12). In the wild, finding and eating nutritionally balanced foods are crucial, and this includes avoiding the potential hazards of consuming unfamiliar food sources (13). Kuo (14) demonstrated that when puppies eat the same food sources for their first 6 months of life they later rejected any novel food source. This was consistent even when the puppies were divested of any food (14). Cheney and Miller (15) also discovered that it often takes several days for an animal to overcome hesitation toward a novel food. Reluctance to consume a new food source is often encountered in the home environment, where owners may find their dog is hesitant when offered a new food type (16, 17).

Domestic dogs are part of the order *Carnivora*, yet despite the name, they are considered omnivorous in terms of their nutrient metabolism (2, 18). These abilities are thought to have come about during the domestication process, when dogs became adapted to a human-associated diet (19). In fact, many free-ranging dogs consume diets high in carbohydrates, and rarely hunt for protein-rich animal-based food sources (4). Omnivores may not only select their food based on its energy content (optimal foraging theory) but also on macronutrient balance (20). Macronutrient balance affects a multitude of variables that play a role in fitness, including growth, fecundity, and disease resistance (21–23). This ability to select a diet that is nutritionally balanced is especially crucial for omnivores, whose food sources can vary, especially when living in the wild (20).

Although vegetables can provide good sources of protein and energy, there is little empirical data concerning the digestibility of individual ingredients by domestic dogs. Vegetable-based ingredients in pet foods have a more consistent composition and macronutrient/micronutrient digestibility than animal-based ingredients (24–26). The use of vegetable-based protein may become more prominent in companion animal food for economic and sustainability reasons, increasing the need for a complete understanding of its food preference. This leads to the pursuit of alternative protein sources in dog food that meet the animal’s nutrient requirements, provide potential health benefits, while maintaining food preference (27).

The objectives of this study were to (1) determine whether dogs display a preference for animal ingredient-based diets when compared with vegetable ingredient-based diets and (2) examine whether dogs experience neophobia to animal ingredient- or vegetable ingredient-based diets. We hypothesized that dogs will demonstrate preference for animal-based protein over vegetable-based protein. Specifically, we predicted that dogs would show greater interest in the food before and after feeding, and feed at a faster rate without distraction. We also hypothesized that dogs will experience the initial effects of neophobia, with those effects declining with time. Specifically, we predicted that dogs will show longer periods of hesitation, reduced interest in the food before and after feeding, and feed at a slower rate with distraction when first introduced to a new diet.

## Materials and Methods

All experiments and procedures were approved by the Animal Care Committee of the University of Guelph, Ontario (AUP# 3543). This behavioral study was part of a larger study focused on the apparent and true mineral digestibility of animal- and vegetable-based ingredient adult maintenance dog food. Dogs were fed two types diets, animal-based ingredient and vegetable-based ingredient, and two diets of different format with a 50% dilution by weight with anhydrous α-d-glucose. This dilution technique, known as the substitution method, allows for a more accurate assessment of the true digestibility of nutrients and accounts for endogenous losses (28).

### Subjects and Facilities

Eight adult beagles (*n* = 8) were used in this experiment. The dogs included two intact males, and six spayed females of similar age (median = 15.2 months, range = 14–24 months) and ranging in body weight (median = 9.6 kg, range = 9.3–11.6 kg). Using G*Power (v. 3.1) (29) and basing effect size on other digestibility trials, where significant differences between digestibility of vegetable/legume and animal ingredient diets were present, we predicted that *n* = 8 was a large enough sample size. Based on previous research and using G*Power, using a two tailed *t* test, an effect size of 3, an α = 0.05 and at different levels of power (1 − β err prob) including 0.8, 0.85, 0.9, an *n* of 8 is calculated, respectively. Client owned dogs outside of the University of Guelph were not included in this study due to the nature of the trial. Dogs were housed in the Central Animal Facility at the University of Guelph, Ontario. Dogs were housed in pairs, with each of the four kennels containing dogs of similar average body weight. The kennels were 121.9 cm × 190.5 cm kennels that were opened with sliding doors to allow for group housing for the majority of day, except during feeding. Kennels also had beds and spring boards located 76.2 cm high. All kennels were in the same environmentally controlled room, with a 12-h light:12-h dark cycle. Dogs were also provided enrichment within their kennels, which included beds and non-edible chew toys (Nylabone). Socialization included walks provided by the researcher and an employee at the Central Animal Facility. This socialization included walks in pairs each day, with walks lasting 20-min, 5 days per week, and 10-min, 2 days a week. This regime was kept consistent for each dog throughout the duration of the experiment.

### Diets

Dogs were exposed to each of four diets in a replicated 4 × 4 Latin square design, with 10-d treatment periods. Ten day periods were selected based on a 6-day adjustment and 4-day collection period for the digestibility trial. There were four periods and four kennels for this design which ensured each dog/kennel received all diets, with each kennel consuming a different diet each period. The Latin square was replicated since two dogs were housed in each kennel and received the same diet. The four dietary treatment diets were (1) animal-based ingredient diet, (2) vegetable-based ingredient diet, (3) vegetable-based diet ingredient diet at a 50% dilution with anhydrous α-d-glucose on an “as is” basis (Sigma Aldrich, St. Louis, MO, USA), and (4) animal-based ingredient diet at a 50% dilution with anhydrous α-d-glucose on an as is basis (Sigma Aldrich, St. Louis, MO, USA) (Table 1).

**Table 1:** Ingredients and nutrient predictions for animal- and vegetable-based diets.

(a) Ingredient profiles of the animal-based diets and the vegetable-based diets		
Ingredients (g/kg diet as is basis)	Animal based	Vegetable based
Fresh beef (liver and trim)	150.0	NA
Fresh potato	130.0	130.0
Corn gluten meal	NA	130.0
Chickpeas and lentils	121.6	120.6
Fresh chicken	120.0	NA
Green and yellow peas	85.0	190.0
Fresh fish blend	85.0	NA
Soybean meal	NA	90.0
Chicken meal	75.0	90.0
Low ash herring meal	50.0	NA
Sweet potato	50.0	50.0
C15066 chicken liquid palatant	25.0	25.0
Chicken fat category 3 spray	25.0	115
Fresh whole egg	20.0	NA
Chicken dry palatant	5.0	5.0
Fresh veggie and fruit blend	5.0	5.0
Enticer B28009	5.0	5.0
Egg powder	4.0	NA
Kelp–Tasco	1.5	1.5
Salt	1.0	1.0
CPF vitamin ADE	1.0	1.0
Natural antioxidant liquid	0.5	0.5
Natural antioxidant dry	0.3	0.3
Acana dog botanical blend	0.1	0.1
Bacteria blend	0.03	0.03

**(b) Nutrient analysis for both animal- and vegetable-based diets**		

**Analyzed nutrient contents (as is basis)**	**Animal based**	**Vegetable based**

Metabolizable energy (kcal/kg)^a^	3,397	3,442
Dry matter %	96	96
Crude protein %	33	33
Crude fat %	11	13
Crude fiber^b^ %	3.4	2.8
Calcium %	0.9	0.5
Phosphorus %	0.9	0.7
Calcium:phosphorus	1	0.7
Omega 3^b^ %	0.54	0.25
Omega 6^b^ %	2.25	3.0
EPA^b^	0.08	0.2
DHA^b^	0.15	0.2
Linoleic acid^b^	2.09	2.90

*^a^Calculated metabolizable energy based on Modified Atwater values*.

*^b^Analysis by Champion Pet Foods*.

Diets were designed to be similar in terms of dryness, texture, kibble size, density, and fallout. Despite having similar macronutrient levels, the animal ingredient-based kibble contained more of its fat internally, while a greater amount of fat was provided externally for the vegetable ingredient-based kibble (2.5% for animal and 12% for the vegetable). The amount of diet provided to each dog (g/day) was determined based on the energy density of each diet and the maintenance energy requirements for individual dogs, which were determined using body weight at the beginning of the study, historical body weight (6-month records previous to study), and historical feeding amounts. Historical body weight values and feeding amounts were used to ensure dogs were consuming enough energy to maintain body weight and that body weight had not changed over the previous 6 months. To ensure equal novelty of the treatment diets, prior to the beginning of the study dogs were fed one commercial dog food of high quality to the same caloric intake as in the current study.

Dogs were fed 95% of their total maintenance energy requirements, in two meals per day, to ensure that there was total consumption of the diet. Dietary energy density was calculated using the Modified Atwater equation and the analyzed macronutrient content of both diets. Diets were extruded at Champion Pet Foods (Morinville, AB, Canada) and formulated to meet or exceed AAFCO nutrient standards. Nutrients were analyzed by near infrared spectroscopy and minerals by inductively coupled plasma analysis (Table 1). Prediction of nutrient content of other key AAFCO nutrients are presented in Table 1, but were not analyzed for. For the diets that were diluted with d-glucose, d-glucose was added to diets on an iso-energetic basis to 50% of the daily caloric intake. Treatment diets were weighed and prepared [addition of glucose and titanium dioxide (Sigma Aldrich, St. Louis, MO, USA)] in advance for each 10-day treatment period. Titanium dioxide was added to each meal to act as an indigestible marker as part of the digestibility study. Warm deionized water was added to each of the fully prepared diets immediately before feeding to prevent the dogs from blowing out any glucose powder, and to ensure mixing and consumption of the titanium dioxide. Kibble was provided using two-cup round, translucent storage dishes (Pyrex, 26.7 cmL × 13.3 cmW × 19.1 cmH). Dogs were fed individually at 0800 and 1300 h each day and had *ad libitum* access to deionized water throughout all four treatments.

### Feeding Behavior

Small camcorders (Sony HD “handycam,” HDR 3.1 megapixel) were set up approximately 60 cm away from kennel doors, and elevated using a 10 L bucket and small tripods to allow for a full view of the kennel. Dogs were acclimated to the cameras and feeding regime for 3-day prior to exposure to their first treatment diet. Dogs were then video recorded during both 0800 and 1300 h feedings, on days 0, 8, and 9 (first and last 2 days) of each treatment period. Thompson et al. (30) found that both shelter and pet owned dogs display consistent preference for food and was one reason that we did not video the feeding experience every day of each of the four periods of the study. Dogs were separated and fed individually with both dogs in each pen receiving a meal simultaneously. The order of feeding was kept consistent throughout the duration of the study. Recordings began approximately 10 s before the dogs were given their meal, and ended approximately 10 s after both dogs finished their meals (all kibble consumed). Videos were then coded for specific behaviors that may indicate their preference for each diet (Table 2). In addition, the duration of feeding was recorded, starting from the ingestion of the first kibble to the last. Rate of consumption was then calculated in seconds per gram for each dog on an as-fed basis. Hesitation prior to feeding was measured as the amount of time (seconds) before the dog took its first bite of food, after the dish was placed on the ground. Number of times dogs focused on other stimuli during consumption was also counted in each feeding bout.

**Table 2:** Ethogram for the behaviors used to analyze the canine feeding experience.

Behavior	Definition
Consumption	Starts with the first bite of food, ends when all kibble has been consumed
Distraction	The dog raises its muzzle out of the bowl, eyes averted, focuses on other stimuli (not food)
Hesitation	Amount of time before the dog begins consumption (s)
Anticipation pre-consumption (0 or 1, absence or presence)	Signs of interest/excitement before eating^a^ Tail wagging Licking air/lips Pushing face through bars Jumping at front of kennel
Interest post-consumption (1, 2, or 3)	Level of interest post-consumption^b^ Little to no interest: dog leaves bowl soon after all kibble is consumed Some interest: dog may lick/sniff bowl/ground after consumption, but loses interest in <10 s Lots of interest: dog licks bowl/ground excessively after kibble is consumed, remains focused on food source until cameras stopped recording (>10 s)

*Consumption, distraction, hesitation pre-consumption, anticipation pre-consumption, and interest post-consumption were recorded to determine preference for animal or vegetable ingredient-based diets, and the effects of neophobia. The specific behaviors were chosen as indicators of food preference or aversion. The presence of each of these behaviors was confirmed after analyzing the video recorded during the 3-day acclimation period*.

*^a^Presence (1) or absence (0) of behavior recorded. Anticipation calculated as sum of four behaviors*.

*^b^Level of interest recorded as one of the three levels (1, 2, or 3)*.

Level of anticipation pre-consumption and level of interest post-consumption were scored per feeding bout. Level of anticipation pre-consumption (10 s before food was presented to the dog) was measured as the presence or absence of four specified behaviors prior to feeding. These behaviors include tail wagging, licking air/lips, pushing face through bars, and jumping at front of kennel. If one of the four behaviors was present, it was given a value of 1 (if absent, it was given a value of 0). Level of anticipation pre-consumption was then calculated as a sum of all four behaviors and given a score of “0 or 1.” Level of interest post-consumption was measured on a scale of 1–3 (1 being little to no interest, 3 being lots of interest). For example, if the dog immediately left the bowl after eating it was given a score of 1, but if it stayed and licked the bowl and/or ground until we stopped video recording then it received a score of 3. If the dog showed interest, but left the bowl before recording stopped it was given a score of 2. Diets which were diluted with 50% with anhydrous α-d-glucose were not included in the analysis of anticipation pre-consumption due to the variability of water added to each meal. However, these diets are essential for the digestibility trial and were included in Section “Materials and Methods.”

All video analyses were completed by the same observer (Meghan C. Callon) who was blinded to the diets that the dogs were receiving. Furthermore, the single observer/coder was trained to code the videos and to improve reliability of the data.

### Statistical Analysis

All statistical analyses were completed using the mixed procedure of SAS (SAS Institute, version. 9.4). Mixed effects repeated measures models were fit assuming the fixed effects of: dietary treatments, format (intact and diluted), day, and time of day. Day was treated as the repeated measure and a compound symmetry covariance structure. The statistical model for each dependent variable included the fixed effects and the interactions. Individual kennels and dog were used as random variables, with treatments being applied to the kennel. Dependent variables were rate of consumption (g/s), distraction (numbers of times the dog focuses elsewhere), hesitation (seconds), level of anticipation (sum of behaviours 1–4), and level of interest after consumption (presence of absence of behavior rating 1–3). When fixed effects were significant for a dependent variable, least square means were compared using the pdiff multiple comparison option. Alpha level of significance was set at 0.05. Differences were considered significant at *p* < 0.05, and as tendencies at 0.05 < *p* < 0.10. Data were expressed as least square mean estimate ± SEM, except for age and BW which were expressed as median and range within tables and figures. Within the text, the differences between least square means, the *t*-value, and *p*-value are presented between contrasts.

## Results

There were no food refusals throughout this study.

For all analyses which included both the intact diets and diluted diets, there were no differences between the two (*p* > 0.10). Therefore, addition of d-glucose and water does not affect feeding behavior, and it is the type of food (animal vs. vegetable), day of feeding, or time of feeding that may have an effect.

### Rate of Consumption

**Figure 1:**
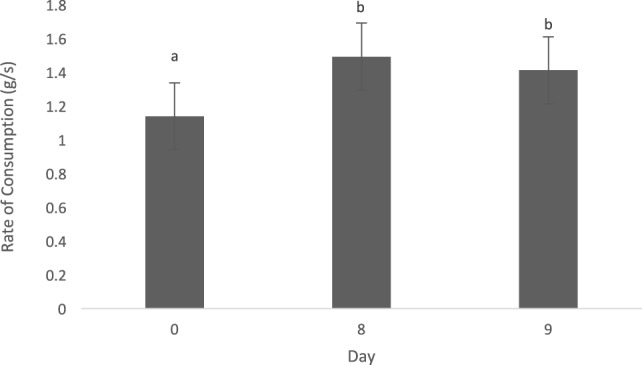
Mean (±SEM) rate of consumption (g/s) for all dogs (*n* = 8) over time (days 0, 8, and 9). Data were pooled across both animal and vegetable diets, as well as a.m. and p.m. feedings. ^a,b^Means with no common superscript differ (*p* < 0.05).

The type of diet (animal vs. vegetable) did not influence the rate of consumption (0.093 ± 0.08, *t* = 1.19, *p* = 0.27). Dogs consumed each diet slower on day 0 of each treatment period, when compared with days 8 (−0.35 ± 0.9, *t* = −3.75, *p* = 0.002) and 9 (−0.28 ± 0.09, *t* = −2.83, *p* = 0.01) (Figure 1). Dogs did not alter their rate of consumption based on the time of day fed (0800 vs. 1300 h; 0.08 ± 0.08, *t* = 0.98, *p* = 0.36).

### Distraction

**Figure 2:**
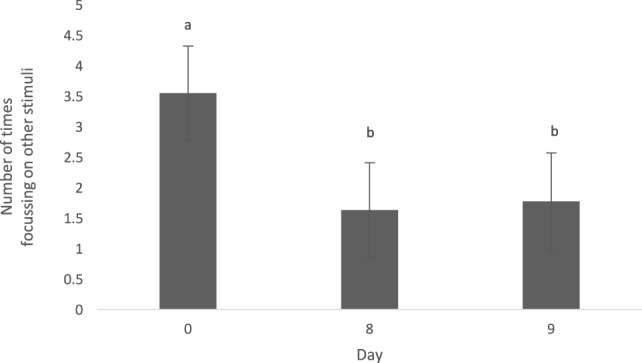
Mean (±SEM) level of distraction was measured as the number of times the dogs focused on other stimuli during the duration of a feeding bout. Data were pooled across both animal and vegetable diets, as well as a.m. and p.m. feedings. ^a,b^Means with no common superscript differ (*p* < 0.05).

The type of diet (animal vs. vegetable) did not influence the level of distraction (0.58 ± 0.45, *t* = 1.30, *p* = 0.24). The number of times the dogs focused on other stimuli throughout a feeding bout (level of distraction) was greater on day 0 when compared with days 8 (1.87 ± 0.54, *t* = 3.48, *p* = 0.003) and 9 (1.81 ± 0.56, *t* = 3.22, *p* = 0.006) (Figure 2). Dogs did not alter their level of distraction based on the time of day fed (0800 vs. 1300 h; 0.59 ± 0.45, *t* = 1.31, *p* = 0.23).

### Hesitation

The type of diet (animal vs. vegetable) did not influence the amount of hesitation (−0.005 ± 0.70, *t* = −0.01, *p* = 0.99). There was no significant difference in hesitation on day 0, than on days 8 (1.48 ± 0.84, *t* = 1.76, *p* = 0.10) but there was a significant difference between day 0 and day 9 (1.91 ± 0.87, *t* = 2.19, *p* = 0.04). Finally, the dogs did not alter their hesitation based on the time of the day they were fed (0800 vs. 1300 h; 0.58 ± 0.70, *t* = 0.83, *p* = 0.43).

### Level of Anticipation Pre-Consumption

The type of diet (animal vs. vegetable) did not influence the level of anticipation (−0.16 ± 0.09, *t* = −1.83, *p* = 0.12). Level of anticipation tended to be lower on day 9, than on days 0 (0.21 ± 0.11, *t* = 1.88, *p* = 0.08). Anticipation was significantly lower on day 9 when compared with day 8 (0.25 ± 0.11, *t* = 2.31, *p* = 0.04). Anticipation also tended to be higher prior to the p.m. feedings compared with the a.m. feedings (−0.18 ± 0.09, *t* = −2.03, *p* = 0.08).

### Level of Interest after Consumption

**Figure 3:**
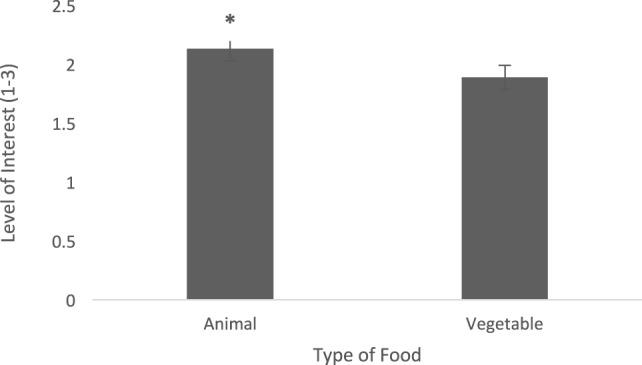
Level of interest post-consumption (±SEM) was measured on a scale of 1−3, 1 being little to no interest, 3 being lots of interest. Data for format of food (water added for glucose dilution diets, vs. dry diets), time of day (a.m. vs. p.m.), and day fed (0, 8, and 9) were pooled. *Indicates a tendency.

**Figure 4:**
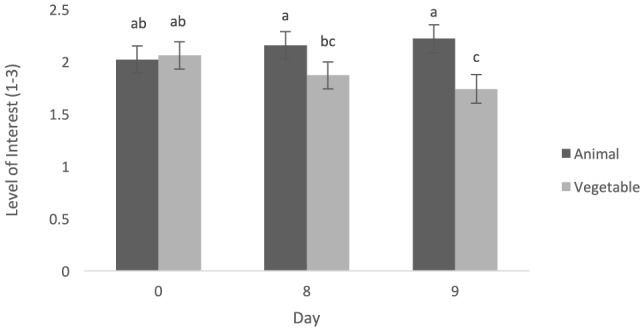
Mean level of interest post-consumption (±SEM) was measured on a scale of 1–3, 1 being little to no interest, 3 being lots of interest. Both data on format and time fed were pooled. ^a–c^Means with no common superscript differ (*p* < 0.05).

Level of interest after consumption was greater when dogs were fed the animal diet when compared with the vegetable diet (0.24 ± 0.09, *t* = 2.89, *p* = 0.02) (Figure 3). Dogs tended to show higher interest (after consumption) when given the animal diet than the vegetable diet on day 8 (0.28 ± 0.14, *t* = 2.02, *p* = 0.06) and had significantly higher interest when given the animal diet than the vegetable diet on day 9 (0.48 ± 0.15, *t* = 3.19, *p* = 0.007) (Figure 4). Dogs showed greater interest in the animal diet on day 9 compared with the vegetable diet on day 8 (0.35 ± 0.14, *t* = 2.46, *p* = 0.027). Dogs also showed higher interest in the animal diet on day 8 compared with the vegetable diet on day 9 (0.41 ± 0.15, *t* = 2.79, *p* = 0.015). On day 0, there was no detected difference in level of interest between dogs fed the animal diets and vegetable diets (−0.04 ± 0.14, *t* = −0.30, *p* = 0.77). Finally, on day 0, dogs showed more interest in the vegetable diet compared with day 9 (0.31 ± 0.15, *t* = 2.15, *p* = 0.049). Time of day also did not alter level of interest after consumption (−0.09 ± 0.08, *t* = −1.07, *p* = 0.32).

## Discussion

This study is the first to use several select canine feeding behaviors (rate of consumption, hesitation, level of interest before, and after consumption) to measure the perceived preference of a food source, and provide a good starting point to develop alternative food preference assessments. These types of behavioral observations are more applicable to the home environment and more accurately represent what consumers/pet owners might encounter when presenting their dogs with a novel food. Indicators of neophobia include longer periods of hesitation, reduced interest in the food pre- and post-consumption, and feeding at a slower rate with distraction when first introduced to a new diet. Overall, the dogs experienced neophobia at the beginning of each treatment period regardless of type of diet (animal vs. vegetable), with those effects declining by days 8 and 9. This suggests that dogs exhibited the disruptive effects of a change in diet. For rate of food consumption, distraction, and increased hesitation prior to eating on day 0, no difference was found between diet types, or in the time of day at which the dogs were fed. Rate of consumption was lower and distraction was highest on the first day of each diet, compared with the last 2 days. Dogs focused on other stimuli more frequently while consuming their meals on day 0. Hesitation was also highest on the first day of each new diet; the dogs took longer to begin eating when presented with each novel food. This suggests that pet owners should not be discouraged if their dog appears to dislike their new food during the first few days of feeding. It is apparent that several days are required for dogs to overcome neophobia. By day 9, the dog’s neophobic responses had diminished. However, due to the fact that feeding behaviors on days 2–7 were not recorded, it is possible that disruptive effects of a novel diet may have decreased sooner. Future research should measure these variables across each day following provision of a new diet to determine what amount of time is necessary for a dog to overcome neophobia.

Level of anticipation pre-consumption was higher on days 0 and 8, decreasing on day 9. This may have been a result of simple hunger. When dogs are awaiting their meals, they may demonstrate excitement, or anticipation, but once presented with the food, they become hesitant (demonstrating neophobia). These results may indicate that the dogs adjusted to the food by the day 9 on each diet, and were no longer anticipating a novel food source. The effect of the presence of cameras was eliminated by acclimating the dogs prior to the experiment. Therefore, anticipatory behaviors on day 9 were not due to the acclimation to cameras, and likely entirely due to dietary acclimation.

Anticipatory behaviors pre-consumption were found to be more frequent prior to the p.m. feeds compared with the a.m. feeds. This may be due to an increase in blood glucose concentrations in the early morning, and a subsequent drop in glucose levels prior to their afternoon meals. This rise and fall in blood sugar in dogs has been reported by Carciofi et al. (31) and is consistent with human research [e.g., Ref. (32)], which found that blood glucose levels rise after 0530 h, and drop significantly 5 h after feeding (often below baseline levels) and this drop elicits hunger.

Since dogs are often considered as primarily meat-eaters, it was expected that they would demonstrate a preference for the diets with animal-based protein, despite more fat being applied to the outside of the vegetable-based kibble. Houpt et al. (33) found that meat-based diets were preferred over a diet composed of maize and soybean meal, suggesting that dogs prefer meat protein to high protein diets composed of non-meat products. Bhadra and Bhadra (4) found that adult Indian free-ranging dogs demonstrated a preference for meat when scavenging. It has also been suggested that dogs will likely find diets lacking any animal-based ingredients less palatable (34). In the present study, the dogs showed a higher interest in the animal-based diets after consumption, although there were no observed differences in feeding rate, level of distraction, hesitation, or anticipatory behaviors between the two diets. Interest post-consumption was evaluated based on the dog’s tendency to lick the ground or bowl after all kibble was consumed, signifying continued interest in their meal. This could imply one of two things: that the dogs found the animal diets more palatable and wanted more, *or* that they found the animal diets less satiating and were looking for more food. Future research should combine these behavioral measurements of the canine feeding experience with satiety hormone concentrations to determine the satiating effects of each diet. In doing so, one can establish whether interest post-consumption is due to acute satiety.

There is currently a lack of data comparing canine preference for animal and vegetable ingredient-based diets that are similar to commercial formulas and the interacting processing (i.e., level of cook, external application of fat or amino acids, etc.). Felix et al. (35) found that dogs demonstrated a preference for diets containing soybean meal, rather than diets containing poultry offal meal. This was determined using a two-pan test where they recorded which food the dogs approached first, and total consumption of the diet over a 30-min test period (35). The current results suggest that the dogs did not have preference for either the animal or vegetable ingredient-based diets with the understanding that more fat was applied to the outside of the vegetable-based kibble, a known palatability enhancement. These results, along with the opposing results by Felix et al. (35), may support the idea that satiation was the main driving force behind the interest post-consumption. This is also supported by Keller (36), who found that plant-based proteins have higher satiety ratings than animal-based proteins. Thus, the dogs may have found the vegetable-based ingredient diets more satiating than the animal-based ingredient diets.

Decreasing voluntary food intake can have a beneficial effect on both health and behavior. A decrease in voluntary food intake may provide a good mechanism to support weight maintenance, allowing dogs to consume fewer calories, while still feeling full. Furthermore, incessant feeding motivation between meals increases behavioral stereotypies, and occasionally even aggression, in dogs (37). Bosch et al. (37) concluded that feeding motivation can be decreased by altering sources and levels of dietary fiber in food, since these can affect both acute and prolonged food intake control. Legumes, such as soybeans, may also provide less variable macronutrient and micronutrient bioavailability and more consistent composition than animal-based ingredients. Indeed, a greater breadth of pulse crops should be investigated beyond the common soybean meal.

There may also be benefits to feeding animal-based protein diets. Based on the results for interest post-consumption, our results suggest that the dogs had a greater preference for the diet containing greater animal-based ingredients, even when there was more fat applied to the outside vs. inside of the vegetable-based kibble. This could be important when developing diets for dogs with more discriminating palates or greater energy requirements. Animal-based protein in the diet also prevents sports anemia in dogs (38). Furthermore, animal-based proteins may allow for higher digestibility of nutrients from the ingredients present, as ingredients in vegetable-based diets may be of poorer protein quality due to binding with other compounds, such as phytate, found in plants and legumes (39) or a poor amino acid balance.

Adding water to canine diets, commonly referred to as “baiting,” is anecdotally reported to increase palatability. However, no relationship was found in the present study between the addition of water in the diets that contained d-glucose and the perceived palatability of a meal. There was no subsequent increase in anticipation pre-consumption, distraction, hesitation, or interest post-consumption with water addition. In addition, research should evaluate the long-term effects of feeding a satiating diet on both feeding motivation and weight control.

## Conclusion

This study provides a good starting point in developing alternative methods of assessing canine food preference that more accurately represent what the consumer might encounter in the home environment. The results of this study suggest that consumers should allow their dog a period of at least 9 days to test out a new diet, before determining whether or not their dog finds it acceptable. These results also suggest that dogs do not have an innate preference for animal or vegetable ingredient-based diets that mimic commercial formulas and that any difference in level of interest may be due to other factors, such as acute satiety, individual ingredients, or processing techniques employed to promote food intake. Further research is required to elucidate the complex variables that influence and predict food preference in dogs and how the owner perceives the feeding experience.

## Ethics Statement

All experiments and procedures were approved by the Animal Care Committee at the University of Guelph, Ontario (AUP# 3543).

## Author Contributions

MC, CC-F, and AS designed the research with major contributions from TD; MC and CC-F conducted the research. MC analyzed data, wrote the paper, and had primary responsibility for final content. All authors read and approved the final manuscript.

## Conflict of Interest Statement

The authors declare that this study received funding from Champion Pet Foods. The funder was not involved in the study design, sample collection, sample analyses, statistical analyses, or interpretation of the data.
